# Genetic parameters for daily milk somatic cell score and relationships with yield traits of primiparous Holstein cattle in Iran

**DOI:** 10.1186/s40781-016-0121-5

**Published:** 2016-10-26

**Authors:** Khabat Kheirabadi, Mohammad Razmkabir

**Affiliations:** 1 Young Researchers and Elite Club, Sanandaj Branch, Islamic Azad University, Sanandaj, Iran; 2 Animal Science Department, Faculty of Agriculture, Kurdistan University, Kurdistan, Iran

**Keywords:** Bayesian approach, Somatic cell score, Yield traits, Relationships

## Abstract

**Background:**

Despite the importance of relationships between somatic cell score (SCS) and currently selected traits (milk, fat and protein yield) of Holstein cows, there was a lack of comprehensive literature for it in Iran. Therefore we tried to examine heritabilities and relationships between these traits using a fixed-regression animal model and Bayesian inference. The data set consisted of 1,078,966 test-day observations from 146,765 primiparous daughters of 1930 sires, with calvings from 2002 to 2013.

**Results:**

Marginal posterior means of heritability estimates for SCS (0.03 ± 0.002) were distinctly lower than those for milk (0.204 ± 0.006), fat (0.096 ± 0.004) and protein (0.147 ± 0.005) yields. In the case of phenotypic correlations, the relationships between production and SCS were near zero at the beginning of lactation but become increasingly negative as days in milk increased. Although all environmental correlations between production and SCS were negative (−0.177 ± 0.007, −0.165 ± 0.008 and −0.152 ± 0.007 between SCS and milk, fat, and protein yield, respectively), slightly antagonistic genetic correlations were found; with posterior mean of relationships ranging from 0.01 ± 0.039 to 0.11 ± 0.036. This genetic opposition was distinctly higher for protein than for fat.

**Conclusion:**

Although small, the positive genetic correlations suggest some genetic antagonism between desired increased milk production and reduced SCS (i.e., single-trait selection for increased milk production will also increase SCS).

## Background

Selection has traditionally focused on production traits. Today it is generally accepted that undesirable genetic associations (i.e., positive) exist between production and health disorders, including mastitis [1–3]. Consequently, it is believed that selection for increased milk production would increase susceptibility to both clinical- and subclinical-mastitis [1, 2, 4]. Mastitis is the most common and the costly disease of dairy cattle (for review, see Heringstad et al. [2]). In addition to the cost of veterinary treatments, decrease in milk yield quality, early culling, extra labor, increased disease risk in the future and replacement costs, mastitis contributes to consumer concerns [2]. Reduced animal welfare, economic losses, and poor milk quality, consequently, form incentives to reduce the incidence of mastitis.

The SCC (concentration of somatic cells) of cow’s milk is an economically important trait in dairy cattle because it can be used both as an indicator of mastitis and as a measure of response to infection [2]. Studies [1, 5, 6] suggest that genetic correlation of clinical mastitis and SCC is moderate to high, implying that selection for low SCC will reduce the incidence of mastitis. In addition, high SCC milk is undesirable for processors because it reduces the shelf-life of consumer milk [7] and diminishes the quality and quantity of milk protein, thereby reducing cheese yields [8]. SCC also has an economic value in their own right in industries where there are milk price penalties for milk supplied for processing with very high counts. High SCC is not only associated with udder health and milk losses, but also negatively affects the longevity [9] and fertility [10] of dairy cows. Thus, incorporation of SCC in selection decisions seems desirable. Commonly, SCC is log-transformed to SCS (standing for somatic cell score).

Possibilities for simultaneous improvement in SCS and yield traits are dependent on the relationship between the traits. Early estimates of the genetic correlation between these traits, especially from the Iran, were few and based on small data sets. Therefore, the objective of the current study is to estimate heritabilities and genetic, phenotypic and environmental relationships between SCS and currently selected traits (milk, fat and protein yield) on test-day basis using fixed regression animal models (FR-AM) and Bayesian inference in the first parity for Holstein cows.

## Methods

A total of 1,078,966 test-day SCC and production traits (milk, fat, and protein yield) records from 146,765 first lactations of Holstein cows recorded from 2002 to 2013 were analyzed. The cows were descendants of 1930 sires (average 76 cows/sire), were distributed over 268 herds (average 547 cows/herd), with age at first calving ranging from 18 to 36 months (in 19 classes). The data were obtained from the Animal Breeding Center of Iran. The test-day yield was limited to ranges of 3 to 75 kg of milk yield, 1.5 to 8 % fact content, 1 to 7 % protein content, and 1 (× 10^3^ cells/mL) to 500 (× 10^3^ cells/mL) SCC; allowable length of lactation was from 5 to 305 d. Test-day SCC were converted to SCS using a base 2 logarithmic function: SCS = log_2_ (SCC/100) + 3 [11]. Only data of animals with at least five animal-observations were kept. A total of 15,517 contemporary groups (herd within year-month of test day) were formed. All known pedigree information of the cows was traced back as far as possible, resulting in relationship matrix of 273,078 animals for the final data set. The structure of the data set after editing is summarized in Table 1.

**Table 1:** Data structure, arithmetic means and standard deviation (in brackets)

Observations (n)	1,078,966
Herds (n)	268
Sires (n)	1,930
Dams (n)	107,372
Cows (n)	146,765
SCS (units)	2.433 (1.149)
Milk yield (kg)	32.82 (7.36)
Fat yield (kg)	1.075 (0.333)
Protein yield (kg)	1.006 (0.239)
Days in milk	151.36 (83.30)
CG^a^	15,517

^a^CG = contemporary group

The analyses were conducted using a multi-trait FR-AM. The model involved the direct additive, permanent environmental and residual random effects. The fixed part of the lactation curve was modeled with a herd by year-month of test-day effect (HYM) and Legendre polynomials with six coefficients for the age-season of calving effect (AS; 76 classes) and with five coefficients for the herd-year of calving effect (HY; 1802 classes). The equation presentation of the model is given by:

$$ {y}_{ijklmn}=HY{M}_{ij}+{\displaystyle {\sum}_{q=1}^6{\beta}_{ikq}{z}_q(t)}+{\displaystyle {\sum}_{q=1}^5{\delta}_{ilq}{z}_q(t)+{\alpha}_{im}+{\rho}_{im}+{e}_{ijklmn}}, $$

where *y*_*ijklmn*_ was the *n*th test-day record of the *m*th cow for a trait *i* (milk, fat, protein yield, or SCS); *HYM*_*ij*_ was the *j*th herd-year-month of test-day effect for a trait *i*; *β*_*ikq*_ was the *q*th fixed regression coefficient for a trait *i* specific to the *k*th age-season of calving class; *δ*_*ilq*_ was the *q*th fixed regression coefficient for a trait *i* specific to the *l*th herd-year of calving class; *q* was the number of covariates; *z*_*q*_(*t*) was a vector of covariates of size *q* describing the shape of lactation curve of fixed regressions evaluated at *t* days in milk; *α*_*im*_ and *ρ*_*im*_, respectively, were the additive genetic and permanent environmental effects of cow *m* for a trait *i*; and *e* was the residual.

The (co)variance components were estimated by Bayesian inference using the Gibbs sampler of the GIBBS3F90 program [12]. A chain length of 200,000 cycles was established, with a burn-in period of 10,000 cycles, and a sampling interval of ten cycles, corresponding to 19,000 samples for subsequent analysis. Convergence of Gibbs chains was monitored by visual inspections of trace plots. The software R [13] was employed for drawing samples from posterior distributions of parameters.

## Result

**Fig. 1:**
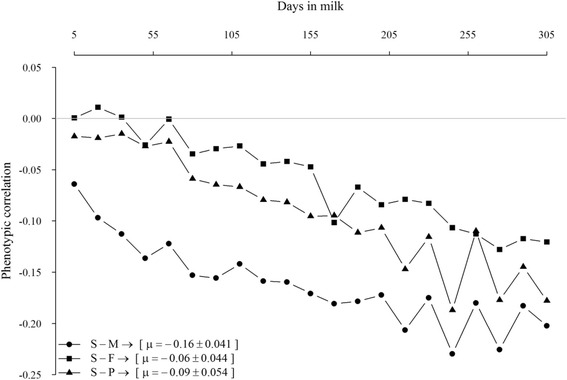
Daily phenotypic correlations between SCS and milk- (S-M), fat- (S-F) and protein-yield (S-P)

Phenotypic correlations between yield traits and SCS on daily basis are in Fig. 1. The relationships were near zero at the beginning of lactation but become increasingly negative as days in milk increased. Average phenotypic correlations between SCS and milk, fat, and protein yields were −0.16, −0.06, and −0.09, respectively.

### Genetic parameters

**Fig. 2:**
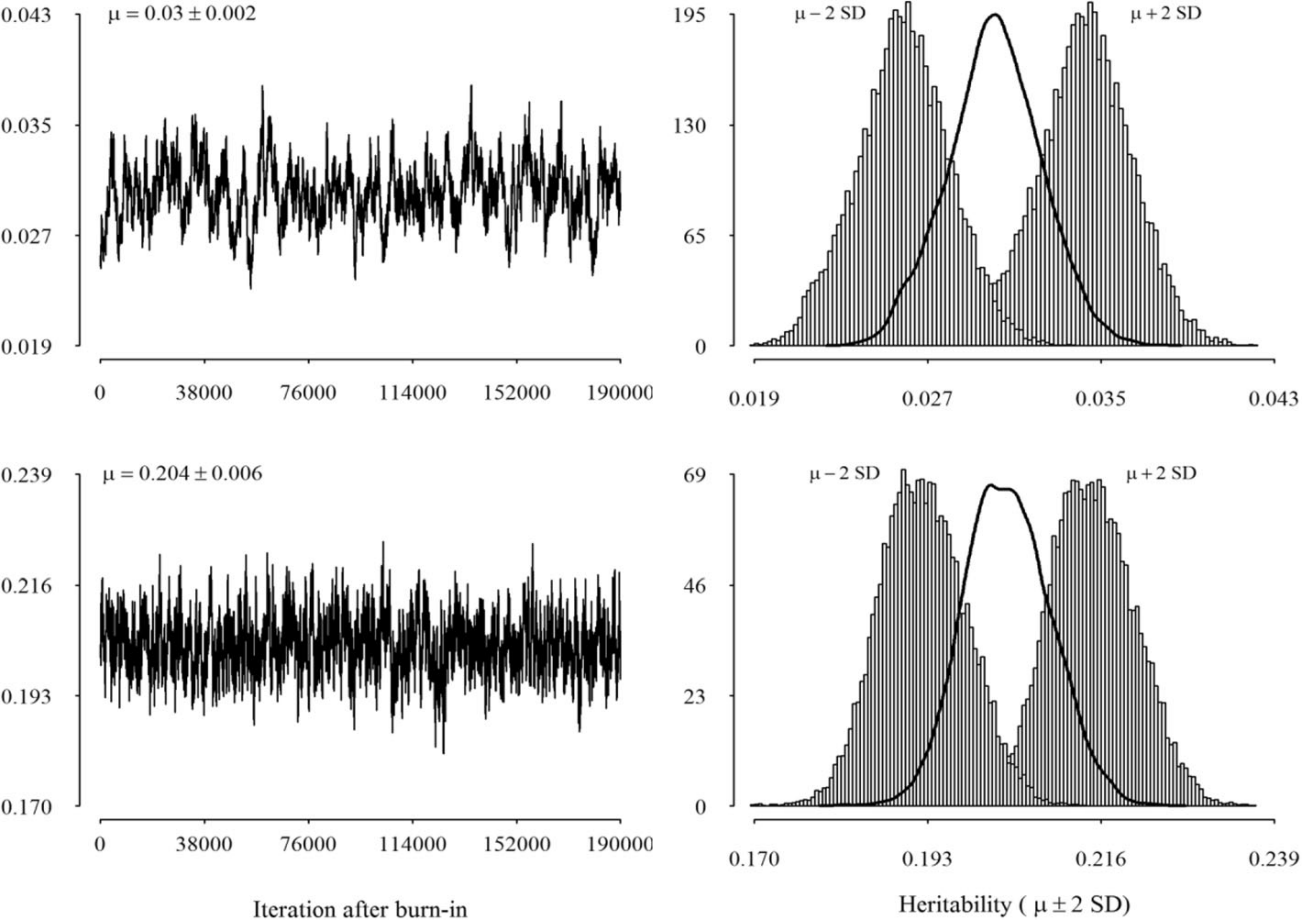
Trace plot (*left panel*) and estimated marginal posterior density (*right panel*) of heritability for SCS (*top panel*) and milk yield (*lower panel*)

Means and posterior standard errors of distributions for variances of random effects are presented in Table 2. Lower genetic variability was observed for protein and fat yields, respectively. Additive genetic variances were always lower than the other variances for all traits. Realizations of heritabilities (mean and 95 % pointwise credible interval) from a Gibbs chain with 190,000 cycles are presented in Fig. 2. For brevity, only trace plots and marginal posterior densities of heritability for SCS and milk yield are shown. Heritabilities for other two production traits (i.e., fat and protein yield) were intermediate to those for SCS and milk yield and were therefore omitted from Fig. 2. The plots indicate that the algorithm mixed well, in spite of differences among traits. In particular, the mixing of the Gibbs sampler was slightly worse for milk, compared to SCS. Means of the posterior densities of heritability for milk, fat and protein yields were 0.204, 0.096, and 0.147, respectively, while Monte Carlo Standard Error ranged from 0.004 to 0.006. Mean of the marginal posterior density of heritability for SCS was low and equal to 0.03, with a 95 % Bayesian credibility region ranging from 0.026 to 0.034 (Fig. 2; upper panel).

**Table 2:** Estimates of means and posterior standard errors (in brackets) of variance components

Trait	Genetic	Permanent environment	Residual
SCS	0.031 (0.002)	0.233 (0.002)	0.761 (0.001)
Milk	7.471 (0.227)	12.633 (0.159)	16.592 (0.025)
Fat	0.007 (3E-04)	0.014 (2E-04)	0.056 (8E-05)
Protein	0.005 (2E-04)	0.010 (1E-04)	0.021 (3E-05)

### Relationships between traits

**Fig. 3:**
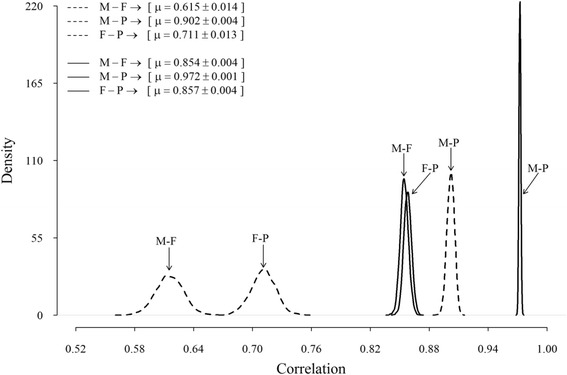
Posterior distributions of additive genetic (*dashed line*) and environmental (*solid line*) correlations between milk and fat (M-F), milk and protein (M-P), and fat and protein yields (F-P)

Posterior estimates of the genetic correlation between the milk production traits were moderate to high (Fig. 3), with the posterior mean (standard error) varied between 0.62 (0.014) and 0.90 (0.004). The highest genetic correlation was between milk and protein, whereas the correlation between milk and fat was the lowest. Estimated posterior environmental correlations were all high, about 0.85 to 0.97, and again the highest estimates were between milk and protein (Fig. 3).

**Fig. 4:**
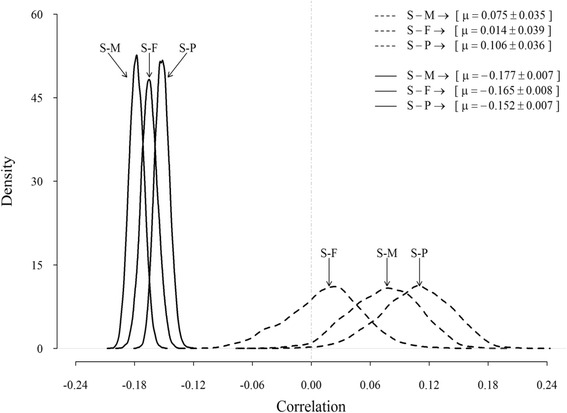
Posterior distributions of additive genetic (*dashed line*) and environmental (*solid line*) correlations between SCS and milk- (S-M), fat- (S-F) and protein-yield (S-P)

Posterior estimates of the genetic correlation between SCS and production traits were low and often symmetric (Fig. 4). Means of the marginal posterior density (highest posterior density of 95 %) of genetic correlations were all positive, but small and averaged 0.07 (0.006 to 0.139), 0.01 (−0.066 to 0.086), and 0.11 (0.033 to 0.171) for milk, fat, and protein, respectively. Posterior distributions of the environmental correlations between SCS and yield traits had a larger mean (−0.15 to −0.18) and a lower standard error (0.007 to 0.008) than corresponding genetic correlations; milk and protein had the largest and smallest correlation, respectively. All these relationships (with the exception of the genetic correlation between SCS and fat yield) can be considered statistically significant, because their 95 % credible intervals not included zero.

## Discussion

Phenotypic correlations between SCS and yield traits were all negative, ranging from 0.0 to −0.24, in agreement with the literature finding that yield traits decreased under mastitis conditions. However phenotypic relationships seemed stranger with advancing days in milk; therefore, the effects of SCS on yields of milk, fat and protein were larger in the late than in the beginning of lactation. This result in contrary to results of most studies [14, 15], which have shown larger correlations during the first 30 d than later. Generally, the negative correlations between yield and SCS are consistent with the deleterious effect of poor mammary health on production [16].

Across production traits, milk yield showed the highest heritability estimates (>0.2). In the earlier study of the Iranian population conducted by Kheirabadi et al. [17], obtained by a random regression test-day model, the daily heritabilities for milk yield were from 0.05 to 0.26 and they were also higher than those for fat (0.03 to 0.12) and protein (0.04 to 0.24). Similar conclusions about heritabilities of milk traits of Holstein cattle in Iranian were reported by Razmkabir [18]. Overall, these results suggest that production traits have enough genetic variation to develop breeding programs. In contrast heritability of SCS was considerable smaller than those for production traits, which in good agreement with other studies [14, 15]. Posterior mean of heritability for milk yield assessed by Wu et al. [19] in the primiparous Norwegian Red cows was 0.18, very close to our results (0.204 ± 0.006), while posterior mean of heritability for SCS was significantly higher (0.12) than our results (0.03 ± 0.002). Haile-Mariam et al. [20] obtained a heritability of 0.07 when test-day records are analysed as repeated measurements. Kheirabadi and Alijani [21] also estimated small values of SCS daily heritabilities for primiparous Holstein cows in Iran (0.03 to 0.07).

As a consequence, standard errors of the estimates of genetic correlations between yield traits were negligible (<0.02). These results imply that yield traits are influenced by similar genes. This corroborates the idea that increasing milk yield by selection would have a positive effect on the other economic traits (referring to fat and protein yields). It seems clear from results in this and other studies [6, 17, 22, 23] that for any pair of traits, genetic correlations were consistently lower than corresponding environmental correlations; this is due to the fact that environmental effects among traits were more correlated than additive genetic effects.

Previous results from studies [6, 19, 23] reporting undesirable genetic correlations of SCS with production traits were confirmed. Mrode and Swanson [1] found, for first lactation, a weighted average genetic correlation between SCC and milk, fat, and protein yields of 0.14 (SE 0.04 to 0.05). Although small, the positive genetic correlations suggest some genetic antagonism between desired increased milk production and reduced SCS (i.e., single-trait selection for increased milk production will also increase SCS). Presumably there is, then, some genetic antagonism between increased milk yield and deteriorate incidence of mastitis as genetic correlations between SCC and mastitis are high [1, 5, 6]. The magnitude of the correlations, however, varied slightly between traits. In agreement with our results, Carlén et al. [6] found the lowest correlation between SCS and fat (0.17 ± 0.04) and the highest between SCS and protein (0.23 ± 0.04) in first-lactation Holstein cows. In general, the relationships between SCS, milk and protein obtained in the present study were in agreement with the results of Yazgan et al. (2010), who found average genetic correlations of SCS with milk and protein equal to 0.07 and 0.14, respectively. Therefore, these results suggest that an increase in somatic cells occurs with an increase of protein content. As shown in Fig. 4, the posterior distributions of relationships between SCS and production traits for the environmental effect were sharper (i.e., less variation), and posterior means had larger absolute values than the corresponding genetic correlation; this reflected the decline in milk yields under mastitis conditions reported in the literature.

## Conclusions

As is usually found in the literature, the genetic correlations between SCS and yield traits were positive, reflecting an antagonism between production and mastitis resistance, whereas the phenotypic correlations were negative, reflecting an unfavorable effect of mastitis on production. This emphasizes the need for including udder health traits in the breeding goal. Results of the present study indicate the presence of low additive genetic variation for SCS in this population of Holstein cows. Therefore, a relatively low genetic progress will be expected following selection. Despite the low estimates of heritability for SCS, the utilization of SCS in order to improve the resistance to mastitis, and in consequence, an increase the profitability of production system, is recommended.

## Abbreviations

FR-AM: Fixed regression animal models
SCC: Concentration of somatic cells
SCS: Somatic cell score
SE: Standard error

## References

[1] Mrode RA, Swanson GJT (1996). Genetic and statistical properties of somatic cell count and its suitability as an indirect means of reducing the incidence ofmastitis in dairy cattle. Anim Breed Abs.

[2] Heringstad B, Klemetsdal G, Ruane J (2000). Selection for mastitis resistance in dairy cattle: a review with focus on the situation in the Nordic countries. Livest Prod Sci.

[3] Vallimont J, Dechow C, Sattler C, Clay J (2009). Heritability estimates associated with alternative definitions of mastitis and correlations with somatic cell score and yield. J Dairy Sci.

[4] Ødegard J, Jensen J, Klemetsdal G, Madsen P, Heringstad B (2003). Genetic analysis of somatic cell score in Norwegian cattle using random regression test-day models. J Dairy Sci.

[5] Emanuelson U, Danell B, Philipsson J (1988). Genetic parameters for clinical mastitis, somatic cell counts, and milk production estimated by multiple-trait restricted maximum likelihood. J Dairy Sci.

[6] Carlén E, Strandberg E, Roth A (2004). Genetic parameters for clinical mastitis, somatic cell score, and production in the first three lactations of Swedish Holstein cows. J Dairy Sci.

[7] Ma Y, Ryan C, Barbano DM, Galton DM, Rudan MA, Boor KJ (2000). Effects of somatic cell count on quality and shelf-life of pasteurized fluid milk. J Dairy Sci.

[8] Politis I, Ng-Kwai-Hang KF (1988). Association between somatic cell count of milk and cheese-yielding capacity. J Dairy Sci.

[9] Sewalem A, Miglior F, Kistemaker GJ, Van Doormaal BJ (2006). Analysis of the relationship between somatic cell score and functional longevity in Canadian dairy cattle. J Dairy Sci.

[10] Rekik B, Ajili N, Belhani H, Ben Gara A, Rouissi H (2008). Effect of somatic cell count on milk and protein yields and female fertility in Tunisian Holstein dairy cows. Livest Sci.

[11] Ali AKA, Shook GE (1980). An optimum transformation for somatic cell concentration in milk. J Dairy Sci.

[12] Misztal I. BLUPF90 family of programs. http://nce.ads.uga.edu/html/projects/programs/Linux/64bit/. Accessed 02 July 2015.

[13] Team RC (2015). R: a language and environment for statistical computing.

[14] Jamrozik J, Bohmanova J, Schaeffer L (2010). Relationships between milk yield and somatic cell score in Canadian Holsteins from simultaneous and recursive random regression models. J Dairy Sci.

[15] Welper RD, Freeman AE (1992). Genetic parameters for yield traits of Holsteins, including lactose and somatic cell score. J Dairy Sci.

[16] Rajala-Schultz PJ, Gröhn YT, McCulloch CE, Guard CL (1999). Effects of clinical mastitis on milk yield in dairy cows. J Dairy Sci.

[17] Kheirabadi K, Rashidi A, Alijani S, Imumorin I (2014). Modeling lactation curves and estimation of genetic parameters in Holstein cows using multiple‐trait random regression models. Anim Sci J.

[18] Razmkabir M. Genetic evaluation of production traits with random regression models in Holstein dairy cattle. PhD Disseration (In persian language). 2011.

[19] Wu X-L, Heringstad B, Chang Y-M, De los Campos G, Gianola D (2007). Inferring relationships between somatic cell score and milk yield using simultaneous and recursive models. J Dairy Sci.

[20] Haile-Mariam M, Bowman P, Goddard M (2001). Genetic and environmental correlations between test-day somatic cell count and milk yield traits. Livest Prod Sci.

[21] Kheirabadi K, Alijani S (2014). Estimation of genetic parameters and genetic trends of somatic cell score in Iranian Holstein cows using test-day records. Iranian J Appl Anim Sci.

[22] Kheirabadi K, Alijani S, Zavadilová L, Rafat SA, Moghaddam G (2013). Estimation of genetic parameters for daily milk yields of primiparous Iranian Holstein cows. Arch Tierz.

[23] Yazgan K, Makulska J, Węglarz A, Ptak E, Gierdziewicz M (2010). Genetic relationship between milk dry matter and other milk traits in extended lactations of Polish Holstein cows. Czech J Anim Sci.

